# Abstract of Proceedings of the Buffalo Medical Association

**Published:** 1869-01

**Authors:** T. M. Johnson

**Affiliations:** Sec’y.


ART. V.—*Abstract of Proceedings of the Buffalo Medical Association.*

TUESDAY EVENING, Dec. 1st, 1868.

The President, Dr. J. R. Lothrop, presiding. Members present—Drs. Lothrop, Little, Cronyn, Rochester, Gay and Johnson.

DR. CRONYN on presenting a morbid specimen for examination, observed that it would be remembered that at a recent meeting of the Association Prof. Rochester mentioned the case of a young man with stricture of the rectum that had been a patient of his, further saying that he thought it cancerous in character. Owing to the unfortunate prejudice against post mortem examination it is seldom we have an opportunity of proving the correctness of our opinions in such fatal cases as in life sometimes admit of a doubt. In this case it will be gratifying to the Professor to find his diagnosis fully verified. The specimen exhibited being the proof. For three months there had been no evacuation from the bowels. The abdomen was enormously distended with flatus, there was a great deal of pain for a time, produced chiefly by various medicines which had been prescribed by one or another, who, knowing nothing of the nature of the disease, for the purpose of acting by catharsis this was allayed easily by morphine. There was no vomiting during the whole period, showing plainly that the sympathies of the rectum differ widely from those of the small intestines, or even other portions of the large. An operation for artificial anus was proposed, but would not be consented to; so nothing further than an amelioration of his condition was attempted; he drank of milk, beef-tea, beef-essence, etc., plentifully and with good appetite, and the assimilation thereof was complete, for on opening the intestines little or no matter was found in them. Here the doctor described particularly the pathological condition of the different organs of the abdomen, found as a necessary consequence of the long continued obstruction, yet notwithstanding how well the stomach and small intestines had performed their functions, the young man died, exhausted, after three months' rectal obstruction. The doctor made several valuable deductions from the whole case as presented.

Dr. Rochester said that he was notified of the *post mortem* but was not able to be present, and was very glad to have this opportunity to see the specimen.

Dr. Lothrop said, that in relation to the rarity of cancer in young persons in addition to what had been already said at a former meeting, he had called to mind two cases of melanotic cancer of the eye and orbit, both of which he saw with Dr. Hamilton, and in one of them assisted to tie the carotid. He had also lately read in Dr. Roger's article in the *American Journal of Medical Sciences*, that of about 30 cases of malignant disease of the scapula, in which removal of the whole or part of the bone was practiced, 10 were under 18 years of age—three or four being under 10 years.

Dr. Rochester said, I think I stated at a former meeting that cancer seems to occur more frequently than formerly. I have in my own experience found more of these cases in young persons than I did twenty years ago, notwithstanding I had better opportunities for seeing this particular disease then than now.

Dr. Lothrop said that when it was stated by friends that there had been no fœcal discharge from the bowels for so long a time as three months, he often thought that they must have been mistaken. He often felt inclined to believe that some fœcal matter must have been passed, in a fluid condition perhaps, which had escaped observation. But whether probable or not, he had known friends to make such statement, which they evidently believed. The late Dr. Wilcox had a case, the patient being an elderly lady, in which there was a tight cancerous stricture at the lower portion of the sigmoid flexure. In that case it was stated that there had been no fœcal discharge for three months. At the *post mortem*

there was found a small cancerous ring, half an inch broad, and not more than one-eighth of an inch in thickness, so narrowing the canal that only liquid could escape through the constriction. The intestine above was dilated into a large pouch, and was filled with fluid material. Even in such cases the intestinal contents from the enlarged pouch above will trickle through and becoming condensed in the rectum below, and be passed away in the shape of a natural evacuation. He had seen one such case, in which there were occasional apparently healthy evacuations, though the constriction was very narrow. This was more likely to be the case when the stricture was high up. When as low as in the specimen presented by Dr. Cronyn, such could not be the case.

Dr. Rochester reported the case of a man admitted to the Hospital of the Sisters of Charity with dropsy. I became satisfied from examination that the patient had enlargement of the heart, and probably granular disease of the kidneys. He was a canal driver and had drank to excess. There was a tumor in the left hypogastric region, large and hard. Thought the tumor might be an aneurism, but from other symptoms there might be abscess of the liver. *Post mortem* examination showed the following condition: Patient, twenty-one years old, five feet eight inches high, weight one hundred and thirty pounds. The kidney weighed fifteen ounces, spleen twenty and a half ounces, the heart twenty-two ounces, and the liver six pounds. He had probably suffered from the heart disease two or three years. There was no obstruction to the circulation; could not account for the hypertrophy of the organs as mentioned.

Dr. Lothrop wished in this connection to speak of a case of enlarged liver which came under his care some years since. In this case a young man presented himself with an enlargement in the region of the liver, which went on increasing until it became very great. There was fever and chills, but no jaundice. Before death it was unmistakably an abscess. After death the right lobe of the liver was found to be an enormous sac containing pus. The left lobe healthy. The abscess contained eighteen pints of yellowish white thick pus. As far as he knew this was the largest quantity of pus in a separate abscess of which there has been any record, though enormous abscesses of the liver are not uncommon.

Dr. Rochester reported a case of sudden death of a young married lady in good health, who was confined November 4th, and attended by Dr. Eddy, who attended her for a week after confinement, and left her as he supposed well. On the 18th of November a messenger came to me in great haste, and said that Mrs. ——— was dead. On going immediately to the house I found that the patient was really dead. She had been quite well for several days, and was quite well in the morning, but at this time, thought she would lie down, and while being helped on the bed fell back and died instantly. *Post mortem* showed rigor mortis well marked. The lungs were found in a healthy condition, heart very small, weight about seven ounces. The cavities of normal size, in proportion to the size of the organ. The walls were normal; structure of walls fatty. The uterus was found in a state of metritis, and containing a grumous matter of offensive odor. She had none of the ordinary symptoms of metritis. Two years ago she had severe typhoid fever and had severe faintings. I think she died of metritis. She had taken no medicine, and there could have been no mistake. I only mention it as a remarkable case of sudden death, resulting possibly from pyemia. The blood was entirely fluid. There was nothing to show that air had entered the veins.

Dr. Lothrop wished to make mention of the case of Christian Gotleib, lately convicted of murder. He had been called to examine him in reference to his being insane. The defence set up was insanity. After several examinations, made with Dr. Hutchins, they came to the conclusion that he was to be classed as an imbecile. He was evidently feeble-minded, but not an idiot, for he was capable of some low prôcess of reasoning; nor was he insane, for there was no evidence of any permanent delusion in any direction or upon any subject. The medical experts did not agree, and Dr. Ring thought imbecility a form of insanity. Dr. Ray so classes it. It was proved that the man was in the habit of self-abuse, and the defence made much of its effect to cause imbecility, and to produce even softening of the brain. It seemed to be the opinion of most of the medical experts that self-abuse was a cause of imbecility. Dr. L. thought this opinion was not well founded. He believed that self-abuse was a consequence and not a cause of weakness of mind. That the imbecility itself, inclining to brutal and indecent impulses, leads directly to the habit. The deteriorating influences of the habit were probably much overstated, even when it is not carried so far as to make idiocy or imbecility chargeable to it. An expert, he thought, should look upon it as an evidence rather than a cause of feebleness of mind.

The subject of masturbation was discussed, all the members present taking part. During the discussion Dr. Rochester said that he was of the opinion that a morbid, deranged condition of the mind or brain was the cause of the masturbation, and that the prevailing opinion among the people and with some medical men that masturbation is the cause of insanity is to a considerable extent erroneous. This was also the opinion of the members present.

Dr. Rochester moved that the fee-bill be so amended as to make the fee for administering chloroform from five to twenty dollars. Laid on the table for one month under the rule.

Typhoid fever and scarlatina were reported as prevailing.

Adjourned. T. M. Johnson, Sec'y.

